# The Royal Boscombe and West Hants Hospital

**Published:** 1909-04-10

**Authors:** 


### THE ROYAL BOSCOMBE AND WEST HANTS HOSPITAL.

H.R.H. Princess Alexander of Teck on Saturday last performed the ceremony of opening the new out-patient department of the Royal Boscombe and West Hants Hospital. H.R.H. the Duchess of Albany had arranged to open the building but was, owing to slight indisposition, unable to undertake the journey, and her place was, at short notice, taken by her daughter. The formal ceremony took place in a marquee before a large assemblage of people; and after having declared the building open the Princess was conducted into the new waiting-hall, where she unveiled a tablet in memory of the late President, Sir Frederick Wills, Bart., to whose munificence the hospital owes so much. The Chairman took the opportunity to announce that Sir Gilbert Wills, the eldest son of the late president, had been nominated to succeed his father in that office. The new out-patient department is a portion of a larger building, which, when completed, will contain the whole of the administrative offices and quarters for the resident medical officers and nursing staff. Separate consulting rooms are provided for the physicians, surgeons, ophthalmic and dental surgeons, and a new dispensary is accessible both for out-patients and for the service of the wards. The new buildings were designed by Messrs. Young and Hall in conjunction with Mr. G. A. Bligh Livesay, of Bournemouth.

